# Multiscale gradients of corticopontine structural connectivity

**DOI:** 10.1038/s41598-025-00886-7

**Published:** 2025-05-12

**Authors:** Paul-Noel Rousseau, Pierre-Louis Bazin, Christopher J. Steele

**Affiliations:** 1 Department of Psychology, Concordia University, Montreal, Canada; 2 Full brain picture Analytics, Leiden, The Netherlands; 3 School of Health, Concordia University, Montreal, Canada; 4 Department of Neurology, Max Planck Institute for Human Cognitive and Brain Sciences, Leipzig, Germany

**Keywords:** Corticopontine, Cerebellum, Pons, Tractography, Gradients, Neuroscience, Computational neuroscience, Neural circuits

## Abstract

The cerebellum’s involvement in a range of cognitive, emotional, and motor processes has become increasingly evident. Given the uniformity of the cerebellar cortex’s cellular architecture its contributions to varied processes are thought be partially mediated by its patterns of reciprocal connectivity with the rest of the brain. A better understanding of these connections is therefore fundamental to disentangling the cerebellum’s contribution to cognition and behavior. While these connections have been studied extensively in non-human animals using invasive methods, we have limited knowledge of these connections in humans. The current work reconstructed the corticopontine projection, the first segment of downstream connections between the cerebral and cerebellar cortices, with diffusion MRI tractography in human in-vivo whole brain data and an independent higher resolution postmortem brainstem dataset. Dimensionality reduction was used to characterize the pattern of connectivity of cerebral cortical projections to the pons as two overlapping gradients that were consistent across participants and datasets: medial to lateral and core to belt. Our findings align with invasive work done in animals and advance our understanding of this connection in humans – providing valuable context to a growing body of cerebellar research, offering insights into impacts of damage along the pathway, and informing clinical interventions.

## Introduction

The historical paradigm of the cerebellum as being a motor control structure has been upended by evidence highlighting its involvement in a range of cognitive and emotional processes^1,2^. The cytoarchitecture of the cerebellar cortex is relatively uniform compared to that of the cerebral cortex, effectively being comprised of the same computational units repeated along its surface^3,4^. This suggests its contributions are at least partially mediated by its pattern of reciprocal connectivity with the cerebral cortex^3^. Accurate characterization of cerebellar connectivity is therefore fundamental to disentangling its roles in cognition and behavior and has potential implications for clinical interventions^5,6^. The first segment of downstream connections between the cerebral and cerebellar cortices, the corticopontine projection, has been extensively studied in non-human animals using invasive methods^7–10^ but almost completely overlooked in humans.

Based on studies conducted in non-human animals, corticopontine projections are characterized by convergence and divergence: disparate cerebral cortical areas project to distinct and fractured patches within the pontine nuclei. Patches are organized in a lamellar fashion, reminiscent of the layers of an onion. Projections originating in sensorimotor areas terminate in a central core interspersed with passing corticospinal fibers and terminations from adjacent cortical regions form external layers^11^. Superimposed on this onion-like organization, cortical areas anterior to sensorimotor cortex project medially whereas more posterior cortical areas project laterally^11^. There is also a rostrocaudal organization, wherein motor areas project preferentially to the caudal pons and association areas to the rostral pons^3^. We can conceptualize these patterns as reflecting three overlapping spatial gradients of connectivity: core-belt, medial-lateral, and rostrocaudal gradients.

Region-of-interest approaches have been used to investigate corticopontine connectivity in humans^12–15^. While these approaches have demonstrated general spatial mappings between the cerebral cortex and the cerebral peduncle (an intermediate segment of the corticopontine pathway) and pons, discrete regions of interest undercut the more nuanced nature of these connections apparent in the non-human animal literature. Other work has applied non-linear dimensionality reduction techniques to diffusion weighted imaging tractography data to delineate fine-grained gradients of connectional topography of different cortical regions^16,17^. Similar approaches have been used in resting state functional connectivity to characterize gradients of functional organization^18–20^. They utilize nonlinear dimensionality reduction techniques (e.g., diffusion-map embedding, spectral embedding) to generate lower-dimensional and overlapping representations of high-dimensional data that encapsulate the dominant modes/gradients of connectivity across space^20,21,17^. Given the nature of corticopontine projections, with their overlapping organizational principles, patterns of segregation and integration, gradient methods are ideally suited to disambiguating these principles in neuroimaging data.

We hypothesized that human corticopontine projections would follow analogous organizational principles to those identified in non-human animals across both scales of dMRI data and on the individual and group levels. Specifically, we expected to find these projections to be organized in a core-belt, and medial-to-lateral fashion^9,11^. Given the inability of dMRI tractography to resolve terminations within the pons^22^, we did not expect to find a rostro-caudal organization. To investigate the organizational principles of the corticopontine pathway in humans we used diffusion MRI tractography and spectral embedding in two complementary datasets: a set of participants from the Human Connectome Project^23^and a single high-resolution acquisition of postmortem human brainstem^24^. The Human Connectome Project data allowed for investigation of individual participant level data and the creation of group averages while the complementary high-resolution sample served as a more granular confirmation of the organizational principles identified in the in-vivo data. With both datasets we (1) generated connectivity fingerprints within the pons that represent the patterns of cerebral cortical projections and then (2) projected the fingerprints to the cortical surface (in-vivo data) or the cerebral peduncle (postmortem data) for visualization. To further compare across the two sets of results we projected the in-vivo pontine gradients along the entire corticopontine tractogram.

## Materials and methods

### In-Vivo dataset

#### Participants

10 unrelated participants (5 females, average age = 29.7) were randomly selected from a dataset used in our previous study^25^ that employed T1w and diffusion weighted imaging data from the Human Connectome Project open-access dataset (www.humanconnectome.org)^23,26,27^. Individual IDs of participants from the dataset will be provided by the authors upon reasonable request. Informed consent for each participant was obtained by the HCP Washington University - University of Minnesota Consortium and all methods were performed in accordance with the relevant guidelines of the Washington University Institutional Review Board (IRB). Structural imaging data were acquired on a 3 T Siemens Connectom Skyra scanner. T1w (0.7 mm iso, TI/TE/TR = 1000/2.14/2400 ms, FOV = 224 × 224 mm) and diffusion weighted imaging (1.25 mm iso, TE/TR = 89.5/5520 ms, FOV = 210 × 180 mm, multiband 3, b-values = 1000/2000/3000 s/mm2, 90 diffusion directions across each b-value). Ten participants were used due to the large computational demands of the tractography procedure implemented in the present study.

#### dMRI Preprocessing

Diffusion weighted images were obtained preprocessed by the HCP data processing pipeline^23^. Preprocessing steps included intensity normalization, distortion estimation and correction and a gradient nonlinearity correction. Diffusion weighted images were aligned to the T1w structural images with a rigid body transformation. MRtrix3 ^29^was used to obtain fiber orientation distribution function (fODF) images that were derived with constrained spherical convolution to perform probabilistic tractography^29,30^.

#### Corticopontine tractography

**Fig. 1:**
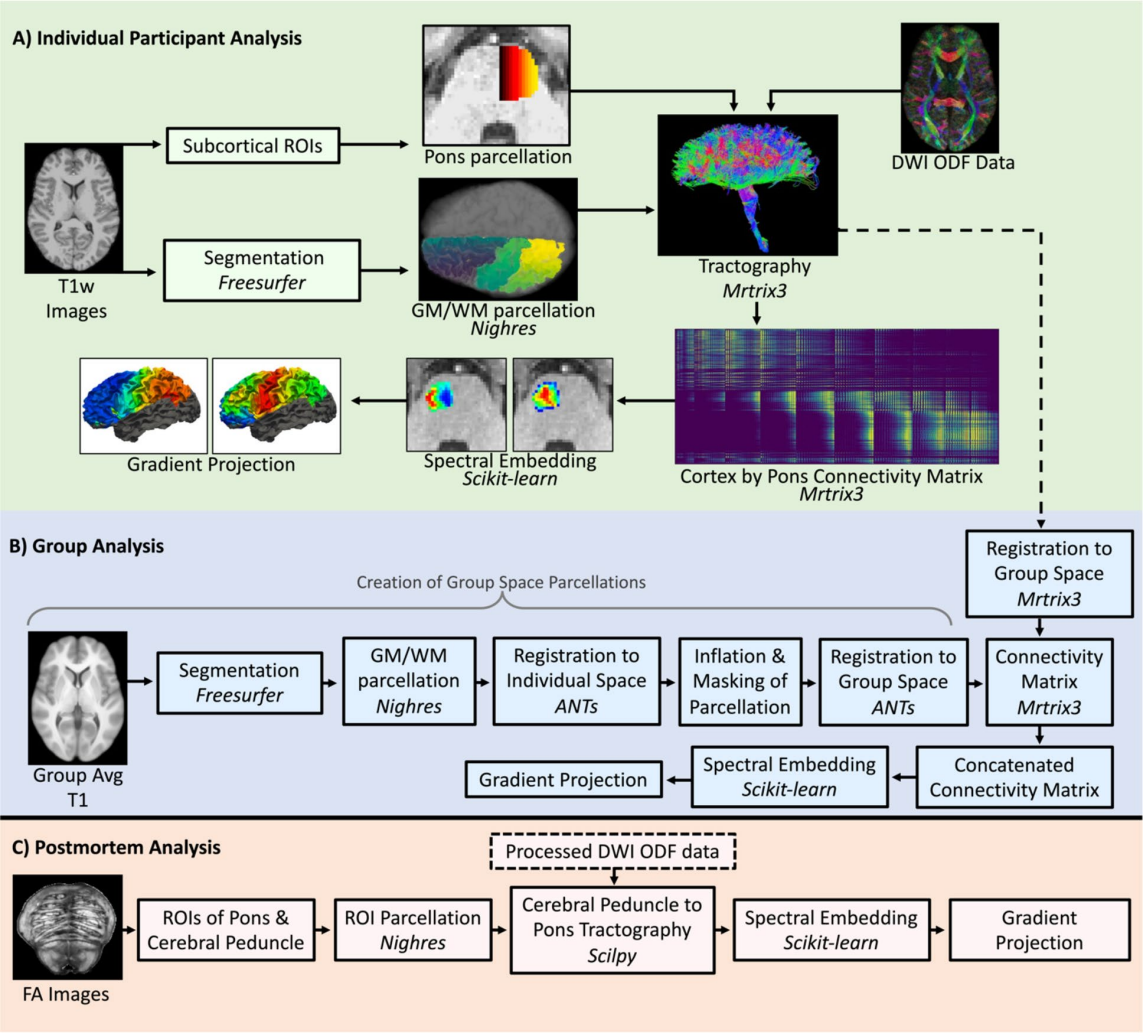
Processing pipelines, including relevant software packages for the individual participant analysis (A), group analysis (B), and postmortem analysis (B).

Our tractography approach (pipeline depicted in Fig. 1a) involved seeding small parcels (i.e., ROIs) distributed across the cerebral cortical surface and performing tractography between each of these parcels and the pons on a parcel-by-parcel basis. This approach was designed to minimize spurious connections, reduce the impact of tractography biases, and to ensure that each area of the cerebral cortex projected a comparable number of completed streamlines directly to the pons. The alternative, wherein the entire cerebral cortex is seeded in one step, would result in an over-representation of streamlines from easier to track regions (i.e. regions with a more linear trajectory to the pons and regions that are closer to the pons)^31^.

## Generation of subcortical ROIs

Generation of Subcortical ROIs.

Hand-drawn regions of interest (ROIs) of the left and right pons and cerebral peduncle for each of the participants were manually created by PNR using MRtrix3’s *mrview* and the T1-weighted and overlaid fODF images^30,28^. *Mrview* was also used to manually delineate exclusion ROIs: a sagittally oriented plane at the midline spanning the corpus collosum (to prevent tracking of fibers to the contralateral cortical hemisphere), coronally orientated plane immediately posterior to the pons (to prevent the tracking of fibers of the middle cerebral peduncle), a small axial region posterior to the middle cerebral peduncle and covering the ascending white matter tracts (i.e. medial lemniscus). An anatomical atlas of the brainstem and cerebellum was used as reference for the delineation of all ROIs of interest and exclusion^32^.

### Generation of cerebral cortex ROIs

The cerebral cortical surface was generated from the T1w images with the Freesurfer pipeline (7.3.2) as presented in detail in our previous work^25^. All analyses were conducted on the left-hemisphere. The boundary between the cerebrum’s grey matter and white matter was converted to a single-voxel thick volume and then subdivided into ROIs of the frontal, somatosensory, parietal, insular, and cingular cortices. These served as the base ROIs that were further subdivided for subsequent tractography. Occipital and temporal cortices were excluded based on previous tract tracing studies in macaques indicating very few white matter projections to the pons from these regions^33^. Freesurfer’s white matter parcellation (*wmparc*) was used to generate ROIs of lobar white matter to be used as exclusion ROIs in the cortical lobar specific tractography. The single-voxel thick cortical lobar volumes were subdivided into small and approximately equally sized and contiguous parcels using Nighres’ *intensity_propagation*, which grew each parcel from voxel seeds distributed uniformly across the lobar volumes^34^. The total number of parcels across the frontal, somatosensory, parietal, insular and cingular cortices was set to 500. A total of 500 parcels was selected to balance between the spatial specificity of the subsequent parcel specific corticopontine tractography, while keeping the computational demands low enough that tractography could complete within a reasonable amount of time. To prevent tracking of invalid streamlines that originate in the seeded parcel and travel to other areas of the cortical white matter surface, a large exclusion ROI consisting of all other white matter surface ROIs was generated for each parcel.

### Cerebral cortex parcel to pons tractography

Probabilistic tractography was performed between each of the cerebral cortical parcels and the left pons, with the cerebral peduncle set as an intermediary inclusion ROI. Tractography was performed with Mrtrix3’s *tckgen*^28^ using the following parameters: iFOD2 algorithm, 1000 completed streamlines, maximum length of 150 mm, step size of 0.625 mm, maximum angle of 45 degrees between steps, initiation, and termination FOD amplitude threshold of 0.1. Tractography was performed independently for the frontal, somatosensory, parietal, insular and cingular cortices and resulted in an approximately equal distribution of streamlines across the cortical surface. This approach allowed for the inclusion of lobe-specific exclusion ROIs to minimize the amount of tracking of spurious streamlines between cerebral cortices and the pons. Frontal parcels included exclusion ROIs of parietal white matter, and parietal parcels included exclusion ROIs of frontal white matter to prevent tracking of superior longitudinal fasciculus fibers. The strict definition of exclusion ROIs was necessary due to the longer maximum streamline length that was used as a requirement to connect more distant regions of the cerebral cortex and the pons.

### Spectral embedding

For each participant we created individual single voxel parcellations of the pons (where each voxel received a unique index label) that were combined with the 500-node cortical parcellations used in the tractography. The resultant node parcellation image was used along with the tractography results (combined across all cortical parcels) to generate a connectome matrix with MRtrix3’s *tck2connectome*. Individual cells in the matrix represented the number of streamlines connecting pairs of parcels (cortex-pons, cortex-cortex, pons-pons). This matrix was further constrained to remove cortex-cortex and pons-pons connections prior to spectral embedding to ensure that our results were specific for cortex-pons connections. In order to mitigate the effect of spurious connections on subsequent spectral embedding the connectivity matrix was thresholded such that pons parcels that received less than 10 streamlines were excluded from the analysis.

Spectral embedding was then used to construct gradients representing the connectivity of the cerebral cortex and the pons, similar to previous work^16,17^. Scikit-learn’s *SpectralEmbedding* function was used to perform spectral embedding on the streamline connectivity matrix^35^. Briefly, the algorithm first transforms the connectivity matrix to an affinity matrix that represents the similarity in streamline connectivity between pairs of pons nodes. Spectral decomposition was then performed on the corresponding graph Laplacian, similar to the procedure adopted by Blazquez Freches and colleagues^16,17^and the top four gradients that represented the dominant patterns of corticopontine connectivity were extracted. Based on our hypothesis that we could identify two gradients within the pons, we analyze and interpret the first two gradients. For completeness we also visually inspected the third and fourth gradients - finding that they contained much more noise and were more inconsistent in their spatial patterns across individuals. For each gradient, each of the nodes within the pons receives a value along a continuous gradient based on the similarity of its connectivity to the cortex compared to other pons nodes, which we remapped to range between 1 and 10 to facilitate display as in previous work. To project gradients back to the cerebral cortex and visualize the spatial correspondence between the pons and cortical gradients, we performed the dot product of the embedding values within the pons and the original pons by cerebral cortex connectivity matrix, and then again remapped the values between 1 and 10. The approach of weighing a connectivity matrix by embedding values has previously been described by others who applied the same general method to gradients derived from functional connectivity^19,36,37^. The final result was that each of the 500 cerebral cortical parcels received a value that represents a weighted average (by number of streamlines) of the embedding values of connected pons voxels. This effectively allows us to visualize spatial changes in corticopontine connectivity patterns across the cerebral cortical surface. The cerebral cortical projections were viewed on the Freesurfer white matter surface for each participant.

### Group analysis

Individual participants’ data were combined to generate group embeddings in a template space based on HCP data^38,39^. In brief, the group template was generated from 1001 participants from the Human Connectome Project^23^. As described by Tremblay and colleagues^38^, a subset of 200 participants were used to generate an initial fODF based template using MRtrix3’s *population_template* function and then individual participant data were registered to template space using *mrregister.* The fODFs were used to drive registration within white matter to prioritize white matter correspondence and ensure that alignment was not unduly influenced by grey matter cortical differences.

For the group analysis in the present study, the ten participant space corticopontine tractograms from the individual participant analysis were transformed to group space with *tcktransform* using the warps generated from the fODF registration (pipeline depicted in Fig. 1b). The average of all ten participants’ T1 weighted images was processed with Freesurfer’s *recon-all* pipeline to generate cortical surface segmentations as done previously at the individual level. The volumetric representation of the cortical surface (including frontal, parietal, insular and cingular cortices) was parcellated into 250 equally sized parcels using the same procedure as in the individual participant analysis. Because of the multiple transforms applied to the parcellation (see below), 250 parcels were used instead 500 to prevent the loss of parcels when transforming between group and individual spaces. A hand drawn ROI of the pons in group space was also created in the same manner as described above, and parcellated such that each voxel was assigned a unique label. In order to obtain a common cortical surface parcellation across participants, the parcellated cortical surface was transformed into individual subject space, dilated such that each voxel took on the value of the closest parcel (Nighres’ *intensity_projection*, 10 mm dilation), and then masked by the cortical gray/white matter interface volume and projected back into group space. The result was individual subject parcellations in group space that were unique to the subject (reflecting their cortical anatomy) but comparable across subjects because parcel indices were maintained and projected to/from the same anatomical location across subjects. For each subject we generated a group space connectivity matrix based on the unique but analogous cortical parcellations and the common parcellation of the pons. The resultant connectivity matrices were summed and thresholded such that pons parcels with fewer than 45 streamlines were excluded from the analysis. The threshold here was set so the resulting connectivity matrix would contain a comparable number of completed pons nodes to that in the individual participants analyses. Spectral embedding and cortical projection were performed on the group total connectivity matrix in the same manner as for the individual subject analyses.

## Postmortem brainstem and thalamus dataset

### Data acquisition and preprocessing

A preprocessed postmortem diffusion imaging dataset of the human brainstem and thalamus from a 65-year-old male was obtained from a previous study by Sitek and colleagues^24^. The acquisition of the original dataset^40^ was approved by the Duke University Health System Institutional Review Board, and all methods were performed in accordance with relevant guidelines. Tissue preparation, MRI acquisition, and preprocessing parameters are described in detail by the authors. In summary, 3D-gradient echo and diffusion weighted Magnetic resonance imaging data was collected on a small-bore Magnex/Agilent scanner. Diffusion-weighted images were acquired with 200 μm spatial resolution (120 diffusion directions at b = 4000 s/m^2^, TR = 100 ms, TE = 33.6 ms, FOV = 90 × 55 × 45 mm). DIPY 0.14 was used to was used to obtain fiber orientation distribution function (fODF) images that were derived with constrained spherical convolution.

### Tractography

Probabilistic tractography was performed between hand drawn ROIs of the left cerebral peduncle and left pons. Due to the incompatibility of the older version of DIPY ODF images with MRtrix3, SCILPY tools (https://github.com/scilus/scilpy) were used to perform tractography on the postmortem dataset (post-mortem pipeline is depicted in Fig. 1c). Seeding was performed in the cerebral peduncle (1000 seeds per voxel, step size 0.1 mm), with an inclusion ROI in the left pons and a large exclusion ROI surrounding the pons and cerebral peduncle. Following the generation of the initial tractogram, the ROI of the cerebral peduncle was then parcellated into 500 approximately equal sized parcels using Nighres *intensity_projection* and the oversampled tractogram was then filtered with MRtrix3 tckedit function such that each parcel included 200 randomly selected completed streamlines. This procedure assured that each parcel in the cerebral peduncle projected an equal number of streamlines to the pons.

### Spectral Embedding and Cerebral Peduncle Projection

To reduce computational load and facilitate comparison at similar spatial resolutions, instead of the voxel-wise pons parcellation used in the in-vivo analysis, the pons ROI was parcellated into 1000 equally sized parcels using Nighres *intensity_propagatio*n. Mrtrix3’s *tck2connectome* function was then used to obtain a connectivity matrix that included the number of streamlines between each of the parcels in the cerebral peduncle and pons. The resultant matrix was constrained to only contain the pons to cerebral peduncle connections (not pons-pons or peduncle-peduncle). Spectral embedding, and projection of embedded values in pons to the cerebral peduncle were performed using the same procedure as in the in-vivo analyses.

### Whole tractogram projection of in-vivo pons gradients

In order to evaluate the correspondence between the in-vivo and postmortem gradients, we also implemented an additional approach to project in-vivo pontine gradients along a group average tractogram. This allows for the inspection of in-vivo gradients along the entire corticopontine tractogram, including in axial slices of the cerebral peduncle that are comparable to the postmortem analysis. We first combined all individual participant tractograms in average space and then filtered them to include only streamlines traversing the cerebral cortical surface, the cerebral peduncle, and the pons. We also used a hand drawn shell around the pons to exclude any stray streamlines exiting the pons towards the MCP. A subset of 100,000 streamlines were randomly selected to reduce computational load and facilitate display. Dipy’s *values_from_volume* function was used to project the average of the embedding values from all voxels in the pons traversed by each streamline onto the streamline for display. The result is a single value per streamline representing the average of the embedding values in the pons voxels it passes through. Mrview was then used to display the tractogram with streamlines color coded according to the range of average embeddings.

## Results

### In-vivo: corticopontine connectivity gradients

**Fig. 2:**
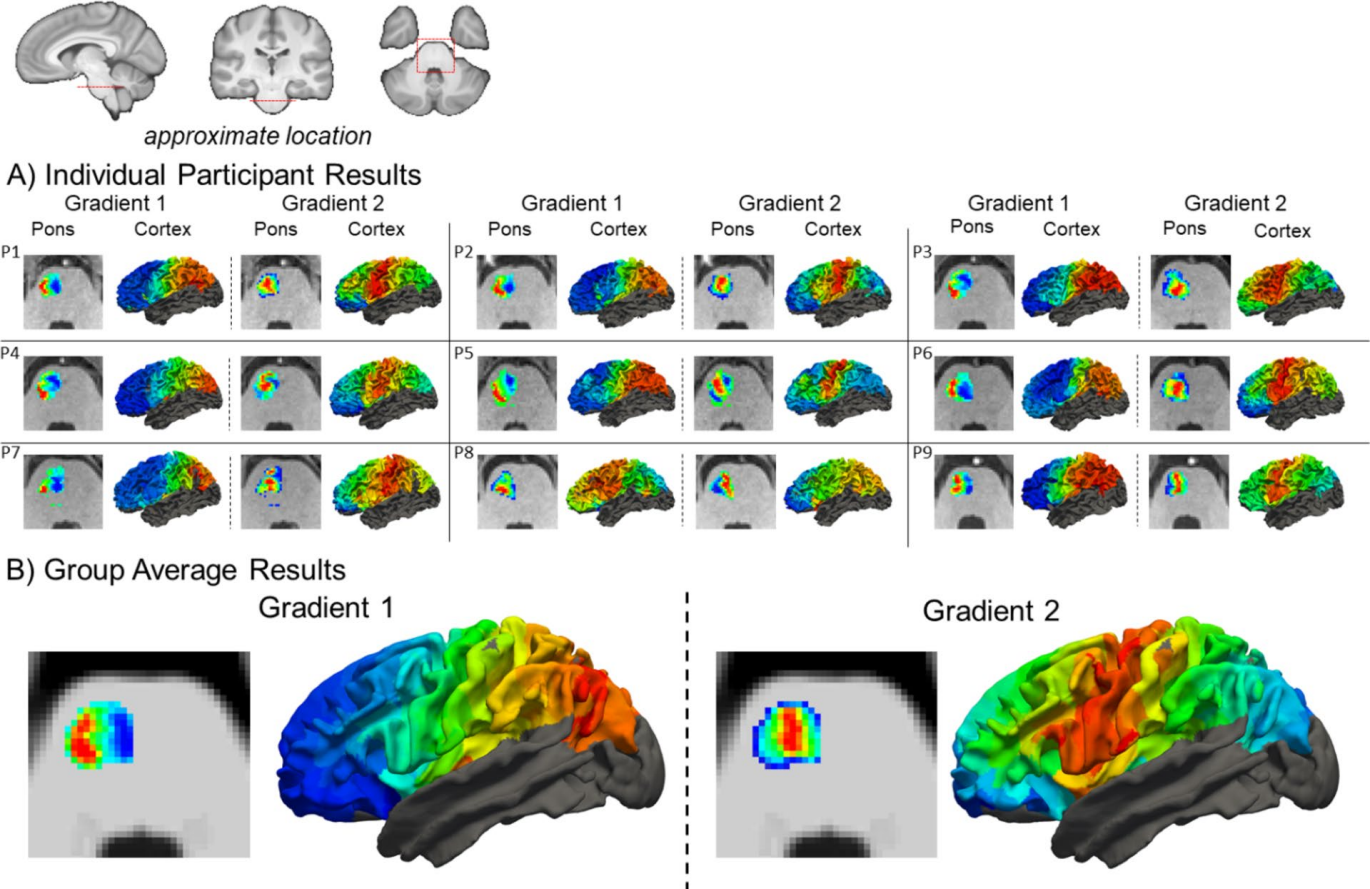
Spectral embedding results and cerebral cortex projection for individual HCP participants (A) and the group average (B). Results show first two components of spectral embedding in an axial slice in the middle of the pons and the associated cerebral cortical projections on the left white matter surface.

Corticopontine tractography and spectral embeddings were successfully completed in 9 of the 10 HCP datasets. In one participant gradient results were discrepant with the rest, specifically there was greater noise evident in the second gradient and it did not contain continuous transitions seen in other participant’s gradients. This appeared to be driven by anomalies in their tractography that resulted in streamlines originating in sensorimotor cortex entering the pons in a more diffuse manner in comparison to other participants. For the 9 successfully completed participants, the first two components reflected a (1) medial to lateral and (2) core to belt gradients when viewed in an axial plane close to the centre of the pons (Fig. 2a). There was remarkable consistency in the gradients across participants. The first component was a medial to lateral gradient for all but one of the participants, where the order of components was reversed. The medial to lateral gradient, did not show a smooth gradation, but was primarily dominated by two zones (medial and lateral) with a small area of gradation between the two. This indicates that the connectivity showed strong segregation between streamlines traveling to the medial and lateral parts of the pons. The core to belt gradient was more variable across participants with some displaying more of a banded gradient, as opposed to more of a concentrically organized gradient (for instance participants 6 and 9). The central area of this gradient corresponds well with regions of the pons occupied by the corticospinal fibres, which is in agreement with tract tracing work in non-human primates^33^.The group analysis reflected very similar medial to lateral and core to belt gradients found in the individual participants (Fig. 2b).

When projected back to the cerebral cortex, the medial to lateral pontine gradient appears as an anterior to posterior gradient (Fig. 2a). In most participants, there is limited gradation within the frontal and parietal lobes and a more pronounced gradation starting at the motor cortex and extending posteriorly. This parallels our observations of the spectral embedding within the pons and may indicate that convergence of cerebral cortical streamlines in the pons results in a loss of spatial specificity. In the cerebral cortex, the core to belt pontine gradient appears as a gradient radiating outward from primary motor cortex. This illustrates good correspondence with the results in the pons, in which the core of the gradient appears to be dominated by corticospinal fibers. The cerebral cortical gradient projection in the group data showed similar results (Fig. 2b), albeit with smoother gradations compared to the gradients of the individual participants. We noted missing data in one of the cerebral cortex parcels in the dorsal aspect of the post-central gyrus. After group registration, this parcel did not have any streamlines connecting to the it the pons, resulting in a null value for the dot-product at this node.

### Postmortem: cerebral peduncle to pons connectivity gradients

**Fig. 3:**
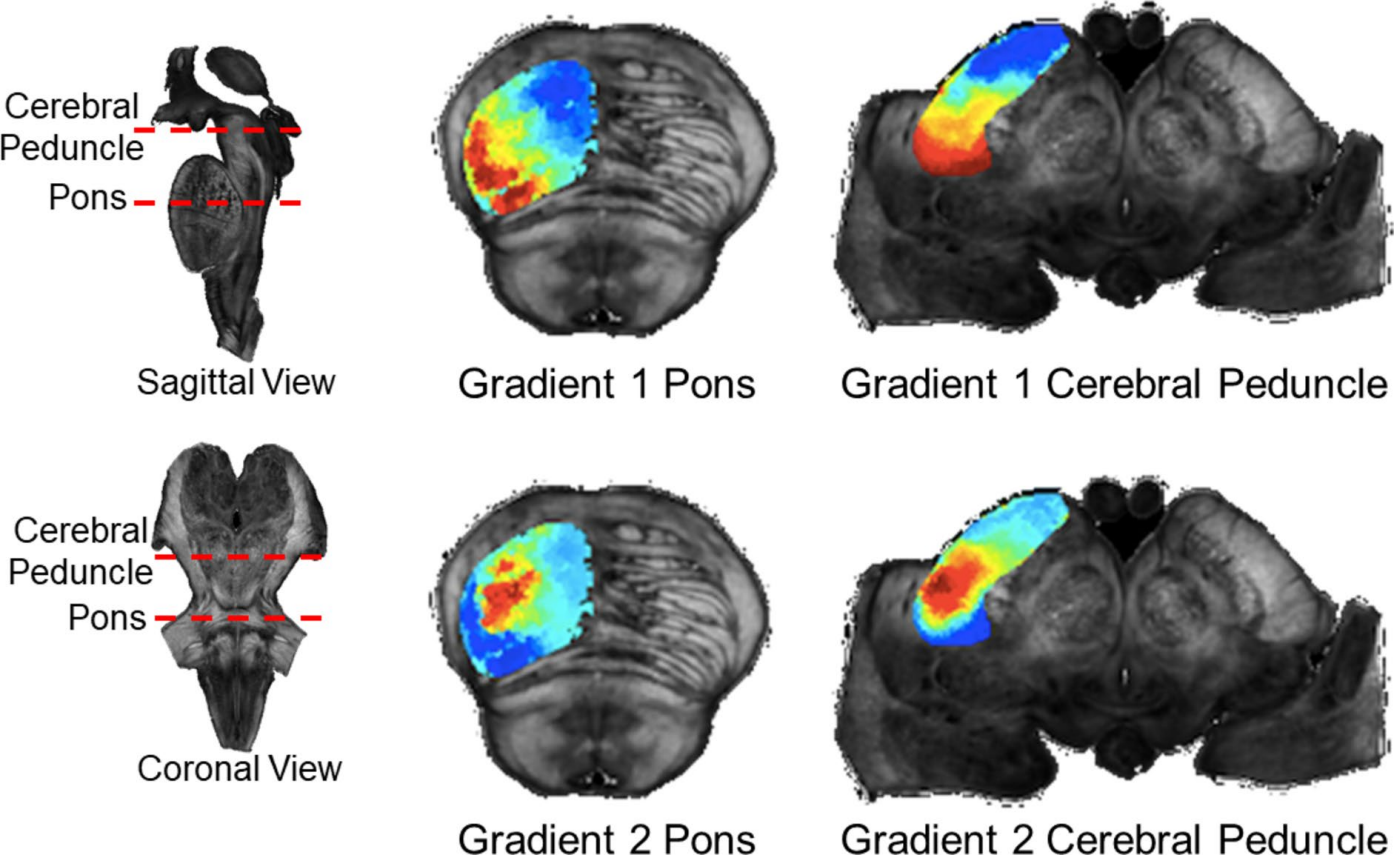
Spectral embedding results and cerebral peduncle projection for postmortem brainstem dataset. Figure depicts first two components of spectral embedding in an axial slice in the middle of the pons and the associated cerebral peduncle projections.

In the postmortem data, we observed two very similar gradients to those identified in the in-vivo analysis (Fig. 3). When viewed in an axial slice in the center of the pons, the first component reflected a medial to lateral gradient, and the second a core-belt gradient. Compared with the in-vivo results, the medial to lateral gradient exhibited finer gradation, and was angled more obliquely. This more oblique configuration is partially the result of the postmortem sample being oriented in such a way that the pons was more closely aligned to the vertical axis than in the in-vivo data. In the core to belt gradient, the core was smaller and more circumscribed compared to the in-vivo results, likely reflecting increased spatial specificity because of higher resolution and decreased partial voluming. When projecting the first gradient to the cerebral peduncle, we observed an oblique anterior to posterior gradient. In combination with the in-vivo results reflecting an anterior to posterior gradient, these findings highlight the lateral twisting of corticopontine fibres as they transition through the internal capsule, the cerebral peduncle, and enter the pons^41,42^. When projecting the second, core-belt, gradient to the cerebral peduncle we observed a core-belt gradient whose centre was in a lateral and inferior portion of the cerebral peduncle – corresponding well with the cortical sensory motor projections previously identified by Ramani and colleagues^15^.

### Projection of in-vivo pontine gradients

**Fig. 4:**
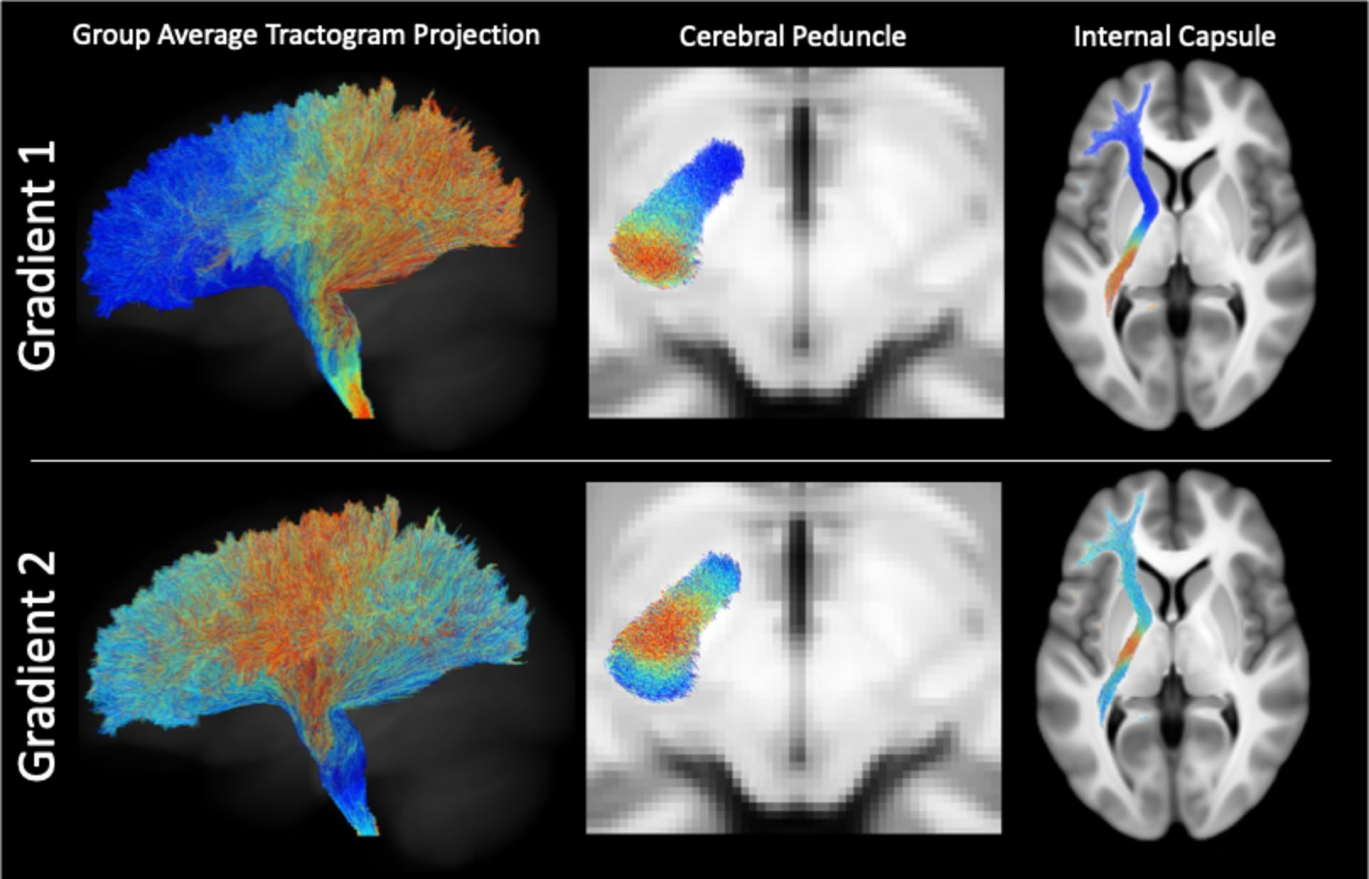
Projection of in-vivo pontine gradients along the corticopontine tractogram. On the left is a three-dimensional representation of the color-coded group sum tractogram. The middle depicts the color-coded gradient projection in an axial slice in the cerebral peduncle, and on the right is the projection in an axial slice of the internal capsule. Streamlines were displayed with 75% opacity and a slab thickness of 1 mm.

The projection of the pontine gradients along the whole tractogram and in axial slices in the cerebral peduncle and internal capsule is depicted in Fig. 4. In the cerebral peduncle, we confirmed a strikingly similar topographical organization of gradients to the postmortem data. With the first gradient oriented on the axis intermediate to the medial-lateral and anterior-posterior axes observed within the pons and cerebral cortical projections, respectively. The second gradient was also remarkably similar to the cerebral peduncle projection in the postmortem data, though exhibiting a somewhat larger central zone that is likely a result of summing across multiple participants’ individual data. When inspecting the projection of the first gradient in the internal capsule, the medial portion of the pons is projected to uniformly from a large segment of the anterior limb of the external capsule that extends slightly past the genu. The central zone of the second in-vivo pons gradient projects to a circumscribed area posterior to the genu, corresponding well with the area of the internal capsule demonstrated to contain passing corticospinal fibres^41^.

## Discussion

We reconstructed the corticopontine pathway using diffusion MRI and tractography in two complementary datasets with different levels of resolution; demonstrating topographic patterns of organization similar to those observed in non-human animal work. Our work is the first to use a data-driven decomposition approach, as opposed to region of interest, to characterize the corticopontine pathway in humans and demonstrates two gradients of connectivity that capture core organizational principles. In the pons, we demonstrate both medial to lateral and core-belt connectivity gradients that are present in both the in-vivo and postmortem high-resolution datasets. In the in-vivo data, the medial to lateral gradient corresponded to an anteroposterior gradient in the cerebral cortex whereas the core-shell gradient corresponded to a cerebral cortical gradient radiating anteriorly and posteriorly from primary motor cortex. We also projected the in-vivo gradients along the entire tractogram to confirm that our two datasets shared the same spatial organization within a region that was common to both, the cerebral peduncle, and explore the organization of connectivity within the internal capsule.

The first gradient, accounting for the largest proportion of the variability in the connectivity data, was organized in a medial to lateral fashion in the pons. In the in-vivo data set, cerebral cortical areas anterior to the central sulcus project preferentially to the medial pons, whereas areas posterior to the central sulcus project more laterally. This configuration (medial to lateral mapping in pons corresponding to an anterior to posterior mapping in the cerebral cortex) may reflect in part the lateral rotation of corticopontine fibers as they traverse the internal capsule and enter the cerebral peduncle. The observed organization in the pons is consistent with and extends the findings of invasive lesion and tract tracing work in macaques that shows frontal cortical areas projecting medially in the pons and parietal areas projecting more laterally^3,11,43^. Within the pons, we observed uniform lateral, central, and medial zones or bands as opposed to a smooth gradation in our in-vivo results. Previous tract tracing work indicates that corticopontine fibers from different cerebral cortical areas terminate in discontinuous patches within the pons^3,43,44^. The patches representing terminations from frontal, sensory-motor, and parietal areas follow the medial to lateral organization that we identified but also exhibit significant overlap^3,43^. Our observations of more clearly demarcated regional projections are likely a function of the lower resolution of the in-vivo dMRI data and convergence of cerebral cortical streamlines on the small cross section in the pons – both resulting in lower spatial specificity than tract tracing. Importantly and supporting this interpretation, the higher resolution postmortem dataset exhibited a smoother gradient that followed the same medial to lateral organization (Fig. 3) and helps to serve as a mesoscale bridge between in-vivo dMRI and tract tracing results.

The secondary core-belt gradient that we observed reflects a funnel-like convergence of projections originating from across the cortical mantle onto the pons. Here we observed a prominent central zone in the pons that, when projected back to the cortex, corresponded to primary motor and somatosensory cortex. The spatial extent of the projection of this central zone was broadly consistent across individual participants (including primary motor cortex in all), however in some individuals it was highly circumscribed on primary motor cortex and in others included somatosensory and/or premotor cortical areas. The position of the projection of this central zone to the cerebral peduncle in the postmortem dataset – along with its link to the primary motor cortex in our in-vivo results – is consistent with previous tractography-based parcellations of the sensorimotor projections^15^. It is likely that the white matter bundles circumscribed within this central zone in the pons consists of both corticospinal and corticopontine sensorimotor fibers. The corticopontine tracing and lesion work in non-human primates has demonstrated that fibers originating in sensorimotor cortex terminate in areas adjacent to corticospinal fibers^11,33^. At the resolutions used in the current study, we were unable to identify terminations within the pontine nuclei or differentiate between corticospinal and corticopontine fibers. Given that corticopontine fibers originate as corticospinal fibers early in development, they are likely in very close spatial correspondence and would be very difficult to differentiate with dMRI^45^.

With dMRI we were limited to detecting gradients along the mediolateral and dorsoventral axes within the pons. Even at 250-micrometer resolution it is not feasible to resolve and differentiate corticopontine white matter and pontine nuclei gray matter with sufficient resolution to reliably detect termination of streamlines. As a result, we were unable to recapitulate another organizational principle within the pons that has been demonstrated in the animal literature: the termination of corticopontine fibers originating in non-sensory motor frontal and parietal areas in the rostral pons (i.e., superior aspect in the human brain) and termination inputs from sensory motor inputs in the caudal pons (inferior aspect)^11,44,46^. However, previous work has indirectly demonstrated this using dMRI tractography between the pons and motor and non-motor areas of the cerebellar cortex^25^, a finding that is corroborated by older post-mortem lesion work in humans^47^.

Our analyses of the corticopontocerebellar pathway with dMRI tractography highlight important limitations in detecting streamline terminations along the rostrocaudal axis in the case of the corticopontine segment, and the mediolateral axis in the case of the pontocerebellar segment^22^. These limitations, in addition to the complex patterns of convergence and divergence of white matter along the whole corticopontocerebellar pathway, emphasize the value of our approach of assessing corticopontine and pontocerebellar segments individually^25^. Reconstruction of the entire corticocerebellar connection in a single step with dMRI tractography (effectively representing this pathway as being monosynaptic), while algorithmically possible, results in a compounding of tractography biases and a lack of spatial specificity of resulting maps. In general, it stresses the value of a more conservative approach to tractography, like the one adopted here, that considers known anatomy while being mindful of limitations of the method^48,49^.

Finally, it is increasingly clear that the pons doesn’t function as a simple relay from the cerebral cortex to the cerebellum, and that it likely serves a computational role that includes filtering and integration of cerebral cortical signals^50^. Our demonstration of overlapping structural gradients and, by extension, organizational principles within the pons, hints at substrates underlying integration of information from disparate cortical areas. Functional work has demonstrated areas in the cerebellum that correspond to the default mode network^51^, which may imply that there is integration of inputs from frontal and parietal default mode areas at some point along the corticocerebellar pathway. An overlap in the two gradients that we have observed, and in frontal and parietal inputs to the pons depicted in the core-belt gradient may reflect this integration at the level of the pons. Applying gradient decomposition to functional MRI data may shed further light on a possible integrative function of the pons. For instance, they may help situate how putative pontine functional connectivity gradients map onto well described cerebrocortical and cerebellar connectivity gradients^18,20^.

## Conclusion

The corticopontine connection, the first step in the downstream pathway connecting the cerebral cortex and the cerebellum, has been studied extensively in non-human animals. Employing two diffusion MRI datasets at different levels of spatial resolution, anatomically grounded tractography, and data-driven gradient methods we are able to demonstrate analogous organizational principles to those seen in animal studies: results that were consistent across individuals and at different levels of granularity. This work informs our anatomical understanding of the corticopontine pathway in humans and lays the groundwork for future research studying more fundamental aspects of this connection and how it may be impacted by different normative and pathological processes.

## Data Availability

Analyses were conducted on publicly available datasets. In-vivo data comes from the Human Connectome Project (https://www.humanconnectome.org). The ex-vivo dataset comes from a prior publication (https://doi.org/10.7554/eLife.48932) and is available at https://osf.io/c4 m82/. Code developed by the authors to perform analyses, as well as final maps in MNI space will be shared on the group’s github page at the time of publication (https://github.com/neuralabc).
